# Whole-genome sequences of marine bacteria presenting the ability to promote the growth of the diatom Phaeodactylum tricornutum

**DOI:** 10.1099/acmi.0.000864.v3

**Published:** 2024-09-27

**Authors:** Rodrigo Martins, Constança D.F. Bertrand, Francisco Quintas-Nunes, Pedro Reynolds-Brandão, Maria T. Barreto Crespo, Francisco X. Nascimento

**Affiliations:** 1 Instituto de Biologia Experimental e Tecnológica (iBET), Apartado 12, 2781-901 Oeiras, Portugal; 2 Instituto de Tecnologia Química e Biológica António Xavier, Universidade Nova de Lisboa (ITQB NOVA), Av. da República, 2780-157 Oeiras, Portugal

**Keywords:** bacteria, genomes, marine, *P. tricornutum*

## Abstract

We describe the whole-genome sequences of seven diverse marine bacteria isolated from Portuguese environments that presented the ability to promote the growth of the model diatom, *Phaeodactylum tricornutum*. The bacterial genome sequences will contribute to the study of genetic and molecular mechanisms involved in diatom–bacteria interactions.

## Data Summary

The Whole-Genome Sequences (WGS) reads and assemblies used can be accessed in the National Center for Biotechnology Information (NCBI) under the following accession numbers: *Muricauda* sp. NFXS6, GenBank: JAGEMG000000000.1, PRJNA714387 (https://www.ncbi.nlm.nih.gov/sra/PRJNA714387); *Thalassospira* sp. NFXS8, GenBank: JAGEMF000000000.1, PRJNA714381 (https://www.ncbi.nlm.nih.gov/sra/PRJNA714381); *Sagittula marina* NFXS13, GenBank: JAGEMH000000000.1, PRJNA714391 (https://www.ncbi.nlm.nih.gov/sra/PRJNA714391); *Sulfitobacter* sp. NFXS29, GenBank: JAGDED000000000.1, PRJNA714103 (https://www.ncbi.nlm.nih.gov/sra/PRJNA714103); *Erythrobacter* sp. NFXS35, GenBank: JAGDEB000000000.1, PRJNA714097 (https://www.ncbi.nlm.nih.gov/sra/PRJNA714097); *Pseudoalteromonas* sp. NFXS39, GenBank: JAGDEC000000000.1, PRJNA714099 (https://www.ncbi.nlm.nih.gov/sra/PRJNA714099); and *Alteromonas* sp. NFXS44, GenBank: JAGDEA000000000.1, PRJNA714087 (https://www.ncbi.nlm.nih.gov/sra/PRJNA714087).

The authors confirm that all supporting data, code and protocols have been provided within the article or through supplementary data files.

## Introduction

The diatom *Phaeodactylum tricornutum* is able to synthesize high levels of industrially relevant molecules such as polyunsaturated fatty acids and fucoxanthin that can be used in a wide range of food and health applications [1]. Importantly, the growth and productivity of *P. tricornutum* can be modulated by beneficial marine heterotrophic bacteria, and these positive interactions may contribute to the development of robust strategies for the *P. tricornutum* commercial-scale cultivation [2].

**Fig. 1.**
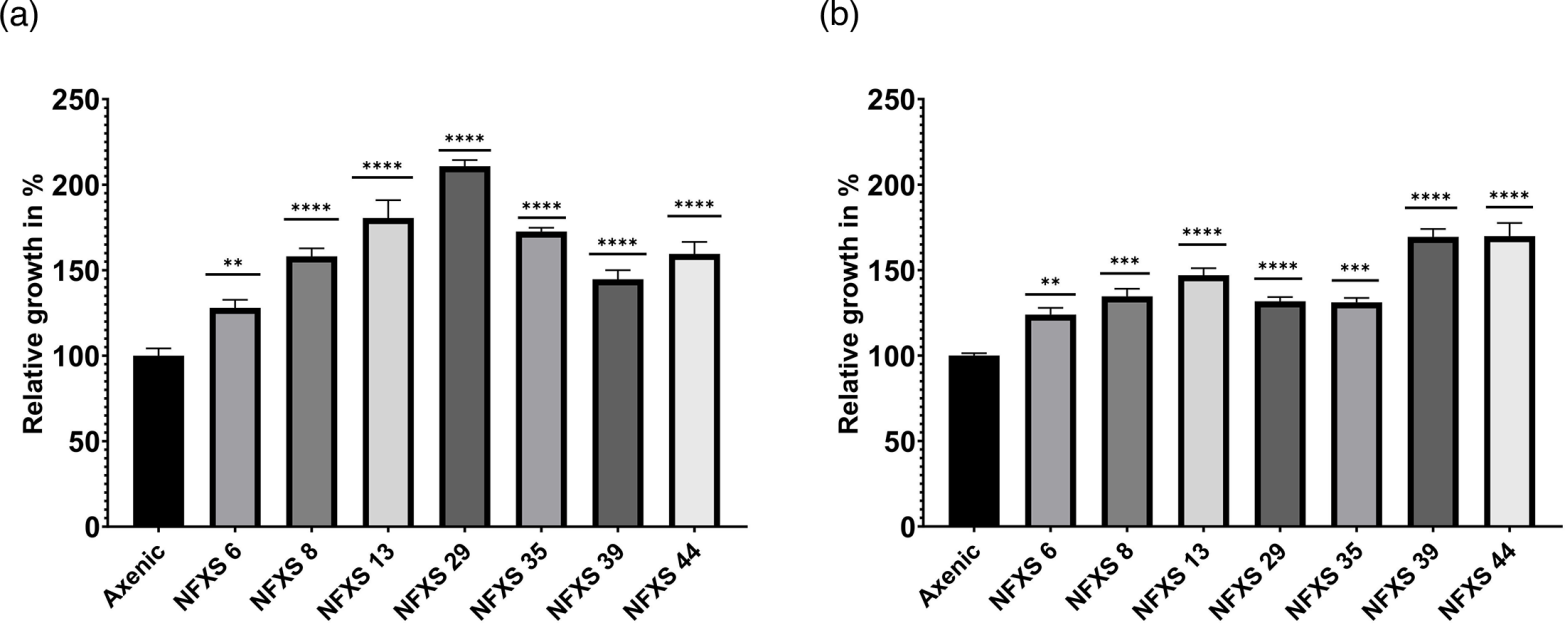
*P. tricornutum* growth-promoting activities (quantified by algal cell counts, cells per millilitre and the relative comparison to the axenic control growth) of the seven studied bacterial strains (*Muricauda* sp. NFXS6, *Thalassospira* sp. NFXS8, *Sagittula* sp. NFXS13, *Sulfitobacter* sp. NFXS29, *Erythrobacter* sp. NFXS35, *Pseudoalteromonas* sp. NFXS39 and *Alteromonas* sp. NFXS44). (**a**) Assay 1. (**b**) Assay 2. ‘*’ represents statistical differences (*P* < 0.05) when compared to the axenic *P. tricornutum* cultivation.

In this study, we describe the whole-genome sequences of seven distinct marine bacteria previously isolated from Portuguese marine environments (Setúbal region) (Table 1) that presented the ability to promote the growth of *P. tricornutum* (Fig. 1). These strains belonged to the classes Flavobacteriia, Alphaproteobacteria and Gammaproteobacteria, which are consistent with the previous reports describing the bacterial communities associated with diatoms [3]. Understanding the genomic properties of marine diatom growth-promoting bacteria is of extreme importance for the development of novel strategies to boost diatom growth and unveil the genetic mechanisms involved in beneficial diatom–bacteria interactions.

**Table 1.** Identification, isolation source and genomic properties of the studied bacterial strains

Strain	Source	PE reads	Genome size (bp)	Coverage X	GC%	Contig no.	N50	CDS
*Muricauda* sp. NFXS6	Marine saltern, Setúbal, Portugal38° 29′ 38.2″ N, 8° 46′ 15.1″ W	1 342 468	4 097 351	156.2	41.5	21	300 994	3617
*Thalassospira* sp. NFXS8	Estuary seawater, Setúbal, Portugal38° 29′ 05.5″ N, 8° 47′ 32.0″ W	2 315 737	5 513 872	202.9	52.9	13	1 589 360	4751
*Sagittula* sp. NFXS13	Marine saltern, Setúbal, Portugal38° 29′ 38.2″ N, 8° 46′ 15.1″ W	1 713 608	5 067 538	163	61.3	53	236 620	4792
*Sulfitobacter* sp. NFXS29	Coastal seawater, Setúbal, Portugal38° 28′ 29.1″ N, 8° 53′ 57.1″ W	651 749	3 947 424	77.7	61.0	30	246 916	3826
*Erythrobacter* sp. NFXS35	Marine saltern, Setúbal, Portugal38° 29′ 38.2″ N, 8° 46′ 15.1″ W	1 364 711	3 380 773	195.6	63.7	11	830 199	3170
*Pseudoalteromonas* sp. NFXS39	Coastal seawater, Setúbal, Portugal38° 28′ 29.1″ N, 8° 53′ 57.1″ W	2 761 720	4 395 030	306	40.8	24	376 643	3841
*Alteromonas* sp. NFXS44	Coastal seawater, Setúbal, Portugal38° 28′ 29.1″ N, 8° 53′ 57.1″ W	2 310 412	5 053 695	222	48.7	16	793 974	4325

## Methods

### Co-cultivation assays

To evaluate the *P. tricornutum* growth promotion activities of the bacterial strains, co-cultivation assays were performed following the protocol described by Bertrand *et al.* [2]. Briefly, the axenic *P. tricornutum* CCAP 1055/1 was routinely cultivated in axenic Guillard’s F/2 media without silica (Sigma-Aldrich, USA). The individual bacterial strains were cultivated in SWPY medium (filtered natural seawater, 5 g l^−1^ peptone, 3 g l^−1^ yeast extract and pH 7.5) for 24 h at 23 °C and 180 r.p.m. and then centrifuged for 7 min at 7500 r.p.m. and 4 °C, and the pellet was resuspended in F/2 medium. The co-cultivation assays were carried out in six-well cell culture plates (VWR, Belgium), receiving a total of 7 ml of F/2 media, *P. tricornutum* cells (final concentration of 1×10^6^ cells ml^−1^) and selected bacteria (final OD_600_=0.02). A total of eight treatments were performed (one plate per treatment and six well replicates), including the axenic control (no bacteria added). Two independent assays were performed for each strain. The plates were incubated in an INNOVA 42R (Eppendorf, Germany) rotary shaker (130 r.p.m.) under light-emitting diode (LED) light at 70 µmol s^−1^ m^−2^ in a 16 : 8-h day/night cycle, at a temperature of 22 °C. The samples were taken 6 days after inoculation, and *P. tricornutum* concentrations (cells ml^−1^) were analysed by flow cytometry using a Muse Cell Analyzer (Luminex, USA). Statistical analysis (ANOVA, Dunnet’s post hoc) was conducted using GraphPad Prism v. 8.2.0.

### Genome-sequencing and analysis

The bacterial strains were routinely maintained in marine agar (Condalab, Spain), and their total DNA was extracted from fresh cultures using the PureLink Genomic DNA Kit (Invitrogen, USA) following the instructions provided by the manufacturer. The extracted bacterial DNA was then sent to Microbes NG (https://microbesng.com/) (UK), where it was processed according to the company’s established pipeline (https://microbesng.com/documents/methods/), and the libraries were constructed using the Nextera XT Library Prep Kit (Illumina, USA), following the manufacturer’s guidelines. However, two modifications were made: the input DNA was doubled, and the PCR elongation time was extended to 45 s. A Hamilton Microlab STAR automated liquid handling system (Hamilton Bonaduz AG, Switzerland) was used for DNA quantification and library preparation. The libraries were then sequenced using Illumina’s NovaSeq 6000 (Illumina) following a 250 bp paired-end protocol. The obtained reads were trimmed using Trimmomatic version 0.30 [4], with a sliding window quality cutoff of Q15, assembled into scaffolds using SPADES v. 3.15.5 [5], which were posteriorly annotated using the NCBI prokaryotic annotation pipeline [6].

## Bacterial strains *P. tricornutum* growth-promoting activities, bacterial genome description and future outlooks

Co-cultivation assays revealed that the seven bacterial strains studied in this work (Table 1) presented the ability to promote * P. tricornutum* growth when compared to the axenic cultivation of the diatom (Fig. 1).

The main characteristics of the final genome assemblies of the seven studied strains can be found in Table 1.

The obtained whole-genome sequences of these marine bacteria will facilitate novel studies regarding (i) the bacterial functional genetic mechanisms involved in *P. tricornutum* growth-promoting activities, (ii) bacterial ecology in the marine environment and (iii) marine bacterial biotechnological properties and their industrial use.

## Abbreviations

LED: light-emitting diode
NCBI: National Center for Biotechnology Information
*P. tricornutum*: *Phaeodactylum tricornutum*
WGS: whole-genome sequences
